# Current diagnosis, epidemiology, and management of interstitial lung abnormalities

**DOI:** 10.3389/fmed.2026.1732754

**Published:** 2026-02-05

**Authors:** Teng Moua, Misbah Baqir, Vasilios Tzilas, Jay H. Ryu

**Affiliations:** 1 Division of Pulmonary and Critical Care Medicine, Mayo Clinic, Rochester, MN, United States; 2 Pulmonary Medicine Department, Medical School, Attikon University Hospital, National and Kapodistrian University of Athens, Athens, Greece

**Keywords:** computed tomography, interstitial lung abnormalities, interstitial lung disease, lung fibrosis, prognosis

## Abstract

Interstitial lung abnormalities (ILAs) are incidental nondependent radiologic findings that may portend early or future interstitial lung disease (ILD), but do not meet specific criteria at the time of presentation. They are subclinical by definition and found more commonly in older ever-smokers undergoing computed tomography (CT) imaging for other indications, including cardiac or lung cancer screening programs. As ILA prevalence increases, driven by an aging population and heightened awareness in younger patients, understanding risk factors for their development and progression has gained recent interest, particularly for optimizing subsequent ILD outcomes. This narrative review summarizes current ILA definitions, epidemiology, risk stratification, and management, while highlighting current challenges and knowledge gaps.

## 1 Introduction

Interstitial lung abnormalities (ILAs) have emerged as a recent area of clinical and research interest, driven by the increased use of computed tomography (CT) imaging for lung cancer (LC) screening, cardiac risk assessment, and systematically assessed population-based cohorts. These incidental, subclinical radiologic findings—now defined and standardized by recent consensus statements from the Fleischner Society (1) and American Thoracic Society (ATS) (2)—represent a spectrum of parenchymal abnormalities that may precede or portend the development of interstitial lung disease (ILD). As identification of ILAs increase, understanding risk factors for their development and potential for progression has become a significant point of focus for optimizing ILD outcomes. This narrative review involved a semi-systematic search of the literature with key words “interstitial lung abnormalities” in several databases, including PubMed (*n* = 199), Web of Science Core Collection (*n* = 119 articles, 110 meeting abstracts), and Google Scholar (*n* = 436, including conference abstracts), returning approximately 390 unique manuscripts with the term in its title or abstract from 1/1/2000 through 8/1/2025 used for this review. These were reviewed and culled according to author-derived subject headings for the manuscript and organized according to type including society or expert guidelines, systematic and narrative reviews, and original cohort or case series describing specific clinical associations or risk factors, epidemiology, and treatment and follow-up. A total of 134 publications were subsequently selected based on relevance to the topic subheadings, with 86 cited. Herein, our review provides an updated summary of current definitions, epidemiology, risk stratification, and management strategies, while also highlighting current challenges and areas of uncertainty.

## 2 Diagnosis and clinical presentation

### 2.1 What are current definitions of ILA?

ILA definitions have evolved over time, moving toward greater standardization and utility (Table 1). The first use of the term was by Washko et al. (3) who described particular radiologic abnormalities and their distribution in a cohort of smokers. This study-specific definition would serve as the basis for subsequent work assessing indeterminate interstitial abnormalities over the next decade. In 2020, the Fleischner Society proposed a standardized radiologic definition and highlighted the clinical uncertainty of ILAs and their potential for progression (1). A practical definition of ILA, independent of pretest probability, was suggested as radiologists often lacked clinical details associated with assessing potential causes and risk for ILD development (2). According to the Fleischner Society, ILAs are non-dependent bilateral changes affecting 5% or more of any lung zone (upper, middle, lower), encompassing combinations of ground-glass opacities, reticular abnormalities, non-emphysematous cysts, honeycombing, or traction bronchiectasis (1). The Fleischner society specifically excluded centrilobular ground glass or nodularity which were part of prior study definitions, as they were often associated with smoking or potentially due to acute inflammatory or infectious processes that may be transient or resolve spontaneously. Focal abnormalities or those with more extended findings potentially consistent with ILD or other causes (heart failure, aspiration, or post-infection) were also excluded. The Fleischner Society added subclassifications into non-subpleural and subpleural fibrotic and non-fibrotic forms (1). A recent ATS guideline retained these criteria but with the specific inclusion of previously excluded high-risk populations (2). Key to both position statements is distinguishing ILA from early ILD, with the latter being considered when clinical and radiologic criteria meet specific ILD diagnoses. The distinction between ILA, pre-clinical ILD, and mild ILD is a current area of contention, recognizing that these classifications may be arbitrary and represent a spectrum of parenchymal disease development and progression (4, 5).

**Table 1:** Evolution of diagnostic criteria and definitions for ILA.

Author(s) and Year	Definition/diagnostic criteria for ILA	Key features
Lederer et al., 2009 (35)	High-attenuation areas in the lung fields of cardiac CT scans, defined as regions with an attenuation between −600 and −250 Hounsfield units, corresponding with ground-glass and reticular abnormalities	Assessed in smokers from the MESA study
Tsushima et al., 2010 (16)	Interstitial changes classified as honeycombing, interlobular septal thickening, ground glass opacities, ill-defined subpleural lines, and combined pulmonary fibrosis and emphysema obtained on prone positioning; extent of disease scored on a scale of 0–30 for involvement of the whole lung	Low-dose CT for LC screening cohort
Washko et al., 2010 (81)	Parenchymal abnormalities suggesting ILD assessed using a qualitative scoring system: 0 = normal, 1 = equivocal for the presence of ILD, 2 = highly suspicious for ILD, and 3 = classic ILD changes	COPDGene study, assessing risk of early ILD in those with smoking history
Washko et al., 2011 (3)	Defined as nondependent changes affecting more than 5% of any lung zone including nondependent ground-glass or reticular abnormalities, diffuse centrilobular nodularity, nonemphysematous cysts, honeycombing, and traction bronchiectasis. Focal or unilateral ground-glass attenuation, reticulation, and patchy ground-glass abnormalities (present in <5% of the lung) were considered indeterminate	First use of the term ILA, established a 5% cut-off
Hatabu et al., 2020 (1)	Incidental, non-dependent CT abnormalities (ground-glass, reticular, lung distortion, traction bronchiectasis, honeycombing, nonemphysematous cysts) involving ≥5% of any lung zone (upper, middle, or lower) in individuals without clinical suspicion of ILD	Introduced non-subpleural, subpleural nonfibrotic, subpleural fibrotic subtypes; specifically excluded centrilobular abnormalities
Podolanczuk et al., 2025 (2)	Nondependent bilateral parenchymal abnormalities on CT (ground-glass opacities, reticulations, lung distortion, traction bronchiectasis, honeycombing) involving ≥5% of a lung zone. Updated to remove exclusion of high-risk populations	Fleischner Society definitions and subtypes retained

### 2.2 How often are ILAs found on incidental lung cancer screening, cardiac, and abdominal CT scans?

Low-dose CT (LDCT) scans often performed for LC screening programs (6–8), coronary or cardiac scans for assessing coronary calcifications (9), and abdominal scans capturing portions of the lower lungs (10), have been associated with incidentally detected parenchymal abnormalities that meet criteria for ILA. The widespread use of these scans has contributed to the increased detection of ILAs, providing opportunities to better understand and distinguish clinically significant cases and underlying risk factors.

The U. S. Preventive Services Task Force recommends annual low-dose CT scans for specific adult populations at higher risk for LC (11), particularly older patients with smoking history. The prevalence of ILAs detected on LDCT varies according to study type and population. One review of over 36,000 LDCT scans over an 18-year period found a prevalence of only 0.3% (12), while another study noted a 2.4% prevalence in their screening cohort (8). One study of 1,382 CT scans using an automated machine-learning algorithm found 8% met criteria for ILA, while 36% were considered indeterminate or potential ILA (13). In asbestos-exposed individuals, LDCT identified ILAs in 32% (14). While validated for detecting ILD, LDCT may be limited in its ability to distinguish fine reticulation from ground-glass opacities or characterize early traction bronchiectasis (15). In one LC screening study, a significant portion of suspected ILAs (e.g., 46%) resolved on follow-up prone imaging, emphasizing the need for follow-up protocolized studies to exclude dependent changes (16). Lastly, ILAs may not only be incidental, but have been associated with worse outcomes in patients with subsequent LC diagnosis (17).

Cardiac or coronary CTs are commonly done as screening and risk stratification for coronary disease, often capturing the surrounding central and lower lung zones. In one retrospective study assessing the potential impact of ambient air pollution on ILAs, 6.4% of 1846 participants with cardiac CTs manifested ILAs with progression seen on subsequent chest CT (18). In the largest study to date of 13,944 cardiac CT scans in 6197 participants, ILAs were detected in 7.3% of cardiac scans vs. 10.5% of full lung scans, suggesting cardiac CTs may underestimate ILA prevalence (9). ILAs have also been detected on the lower lung cuts of abdominal CTs, which often capture portions of the adjacent lower lung parenchyma. One study systematically assessed the prevalence of ILAs using a large, unselected sample of 21,118 patients undergoing abdominal or thoracoabdominal CT imaging. ILA was detected in 1.7%, with 43.9% of these not being documented on original radiology interpretations (10). Routine use of lung window settings for both abdominal and thoracoabdominal CT scans is suggested for better recognition of ILAs (10).

### 2.3 What is the reported incidence or prevalence of ILAs across general populations and specific subgroups?

Reported prevalence of ILAs vary due to differences in study definitions, population characteristics, and age range of examined cohorts. Pooled ILA prevalences have been reported as 7% in the general population, 7% in lung cancer groups, and 26% in familial cohorts (5). One study from an LC screening population reported an ILA prevalence of 9.7% (86 out of 884 participants), with an additional 11.5% having indeterminate findings (19). Similarly, ILA prevalence of 9.7% was found in a large prospective cohort of over 29,000 Swedish participants, including a little over half who were never-smokers (20). Among smokers, ILA prevalence ranges from 4 to 9% (5, 21, 22). The highest risk group continues to be first-degree relatives of those with familial pulmonary fibrosis, reported as 24–26% (23, 24). One meta-analysis described geographical prevalences of ILAs, noting a pooled prevalence of 12.4% in the United States, 7.1% in Europe, and 8.9% in Asia (25). A multicenter cohort study from Korea reported ILA in 3% of individuals over a 4-year period (26). Prevalences may be underestimated if individuals over 75 are excluded (2) or there is a higher proportion of younger patients (<55 years) being screened (27).

### 2.4 What are presenting clinical and demographic characteristics associated with ILA?

Individuals with ILA often present with distinct clinical characteristics and demographics, which include a higher proportion of older individuals and smokers, as well as increased comorbidities (5, 26, 28–32). This may reflect greater frequency of screening or diagnostic testing in such patients for unrelated reasons, rather than associated characteristics being mechanistically involved in ILA development or progression. Patients with ILA are generally older with prior studies showing an association between ILA and increasing age (6, 9, 27, 29, 30, 33, 34) as well as male predominance (30), though two cohort studies have reported a female predominance (29, 33). ILA is also associated with smoking history (9, 34, 35), occurring in 4% of smokers compared to 2% of non-smokers in one cohort (26). Another study involving smokers found ILAs in 8% with impact on clinical outcomes including more restrictive impairment (42% vs. 30%) and less radiologic emphysema (14% vs. 30%) (3). Increasing number of pack-years has also been associated with ILA (36 vs. 15 pack-years, *p* < 0.001) (26).

Lastly, patients with ILA tend to have more comorbidities, likely due to older age at presentation (28, 33). One study described an association of obstructive sleep apnea (OSA) with ILA (described as high attenuation areas, occurring in 11.3% per 10 years) after adjusting for confounders (age, sex, smoking history, height, and weight) (36), particularly greater sleep fragmentation correlating with increased parenchymal abnormalities (37). Insulin resistance has also been associated with increased ILA risk (adjusted odds ratio (OR) 1.35 [1.05–1.74]), as described in one large cohort study of over 800 patients (38). Prior studies have found higher body mass index (BMI) and adiposity being associated with greater prevalence of ILA and risk of progression (29, 39). Specifically, higher amounts of visceral and pericardial adipose tissue were linked to early lung injury and lower lung function, with inflammatory mediators such as interleukin-6 and leptin partially explaining this association (40). While age and background prevalence of comorbidities may be confounding, most studies were large retrospective cohorts with multivariable adjustment for baseline characteristics including age, BMI, and smoking history, suggesting potential independent association of specific findings with ILAs.

### 2.5 What is the prevalence of ILAs associated with lung cancer?

Previous studies have reported higher ILA prevalence among those screened for or diagnosed with LC. A 2025 meta-analysis of 24 studies involving 7,859 patients with LC found an unadjusted ILA prevalence of 17%; 9% after correction for study heterogeneity and population differences (41). One study reported an ILA prevalence of 3.9% in their LC screening program, with 40.7% progressing to ILD within 5 years (42). Reported ILA prevalences may also vary according to LC stage, with one study highlighting 3.9% in those with stage IV non-small cell lung cancer (NSCLC) (43) and 21.7% in stage I and II disease (44). Two other studies reported a prevalence of 9.5% in patients with stage I NSCLC (45, 46). ILAs are also associated with increased morbidity when associated with LC diagnosis, particularly complications related to treatment and survival outcomes (17). LC associated with ILAs is characterized by male predominance, older age, heavier smoking history, and increased incidence of squamous cell carcinoma (17). ILAs were an independent risk factor for five-year LC mortality in one case–control matched study (52% vs. 76% survival) (47), and often associated with greater post-operative complications, radiation pneumonitis, and adverse effects to immune-checkpoint inhibitor therapy (48–50).

### 2.6 What is the prevalence of ILAs associated with COPD or emphysema?

ILAs appear to occur at higher rates in patients with chronic obstructive pulmonary disease (COPD) or radiologic emphysema compared to the general population. A systematic review of 11 studies found the prevalence of ILAs ranging from 6.5 to 28.4% (51), dominated again by male sex and higher pack-years. Specific studies have shown prevalence rates as high as 10–14% (52) and 40.7% (53), respectively. One study found ILAs co-occurring with COPD in 40.5% of patients (54), while another reported ILAs developing in 7% of COPD patients over a 5-year period (52). A large cohort study assessing active smokers reported an ILA prevalence of 8%, associated with more restrictive impairment and less radiologic emphysema (3). Specific risk associated with ILAs and the development of future ILD or LC is unknown, with likely only a small percentage going on to meet clinical and radiologic definitions of combined pulmonary fibrosis and emphysema (55), where more extensive fibrosis is often required.

### 2.7 Are there genetic or familial risk factors associated with ILAs and their progression to ILD?

Familial cohorts show a much higher prevalence of ILAs (up to 26%) and increased risk of progression compared to general populations (5, 23). Genetic and familial risk factors—most notably the MUC5B promoter variant (56) and pathogenic variants in telomere-related genes (57)—may be associated with both the presence and progression of ILAs and ILD. In one cohort study, ILAs were detected in 31% of first-degree relatives of patients with idiopathic pulmonary fibrosis (IPF) with eventual ILD diagnosis in 58% (24), though there were no clinical differences between cases associated with sporadic or familial disease. Another study found 15% of unaffected family members in those with familial disease went on to develop ILAs, while 65 and 77% of those with mild and moderate ILA presentations, progressed over a mean follow-up of 6.2 years (58). Data from the Framingham study found increased copies of abnormal MUC5B alleles were associated with increased risk of developing ILAs (OR 2.8 [2.0–3.9]) or lung fibrosis (OR 6.3 [3.1–12.7]) even after adjustment for age, sex, BMI, and smoking history (56). A genome-wide association study of several population-based ILA cohorts found overlap between ILA and IPF-related loci, including MUC5B (59), while another population-based study identified additional loci associated with related mechanisms of fibrosis including cell adhesion and glycosylation in those with ILA (60). Mean telomere length was associated with a two-fold greater risk of developing ILAs in one study reviewing several population-based cohorts (57). Polygenic risk scores combining genome-based assessments and specific gene abnormalities have been shown to be predictive of ILA and ILD development (61). While the clinical utility of genetic testing lies in identifying individuals at highest risk (62), there is no consensus on optimal screening intervals or cost-effectiveness, with routine genetic screening not broadly recommended outside of high-risk familial settings (4, 63). The current ATS guideline recommends chest CT screening for ILAs or early ILD in adults ≥50 years with a first-degree relative with familial fibrosis, but not routine genetic testing or screening in those found to have ILAs (2).

### 2.8 Are there environmental or occupational exposures associated with ILAs or their progression to ILD?

Rice and colleagues (18) reported the only systematic assessment of potential ambient air pollution and risk of ILAs. Using data from the Framingham study, they assessed the distances of participant homes to major roadways and five-year ambient fine particulate, elemental carbon, and ozone levels using temporospatial models and found increased risk of ILAs (OR 1.27 [1.04–1.55]) in those with greater exposure (18). Self-reported exposure to mold, birds, aluminum smelting, and lead, were also associated with greater risk of ILA development in a cohort of unaffected first-degree relatives with familial fibrosis (34). Occupations associated with exposure to environmental pollutants may contribute to increased risk of ILAs, though no studies have identified particular settings or occupations (20). Studies involving environmental or occupational exposures are epidemiological and associative but difficult to interpret due to challenges with external validity.

In summary, reported prevalence and progression rates of ILAs vary widely across studies, reflecting heterogeneity in study design and population, imaging protocols, radiologic definitions, baseline exclusion of ILD, and duration of follow-up.

## 3 Prognosis and management

### 3.1 What percentage of ILA progress or develop ILD?

ILA progression rates vary widely across studies, reflecting differences in methodology, follow-up periods, and assessed populations, with studies reporting radiologic progression rates from 20% over 2 years to 73% over 5 years (5) (Table 2). Notably, most studies described ILD diagnoses as being made by pulmonologists after consultation or referral, not by radiologic definitions alone or by which specific criteria. One study found only 12 out of 94 patients with ILA (12.8%) progressed to ILD (defined clinically by pulmonary providers as IPF vs. non-IPF), despite 80% having visual progression (26). Another study involving 41 subjects reported 10 patients (24.4%) progressing to ILD as defined by formal pulmonary referral, with a mean time to ILD diagnosis of 4.47 years (8). In contrast, approximately 43% of patients with ILAs initially detected on LDCTs showed progression during a mean follow-up of 7.3 years, with 70% subsequently diagnosed with ILD (12). In another study, the median time to ILA progression was 3.2 years, with 81% having radiologic progression after excluding those with initial ILD (64). The pooled progression rate for ILA was 31.0% for shorter follow-up periods (less than 4.5 years) and 64.2% for longer follow-up periods (greater than 4.5 years) (25). Lastly, ILAs with more extensive radiologic findings may require immediate clinical assessment to exclude symptomatic or functional abnormalities associated with underlying ILD. Those with greater than 10% parenchymal involvement are often more likely to be classified as ILD on initial assessment (89.3% vs. 46.7%), despite more respiratory symptoms being reported in those with ILA compared to initial ILD (65). In a screening population out of the United Kingdom, 65% of patients with radiologically suspected ILA were subsequently diagnosed with ILD after clinical review (42). In another cohort of 443 patients with initially suspected ILA, 54% (239 individuals) were found to have ILD (66). In summary, clinical diagnoses of ILD appear to be loosely described and reference expert clinician judgment rather than clear symptomatic or functional thresholds in terms of transitions from ILA to ILD.

**Table 2:** Percent and rate of ILA progression.

Author, Year	Population/Setting	% Progression to ILD or radiologic progression	Timeline/Rate	Notes/Definitions
Park et al., 2023 (64)	Screening cohort, median age 62 years	80.5% with ILA had radiologic progression; 17.3% progressed to UIP-like ILD	Median of 3.2 years for ILA progression; 11.8 years for ILD with UIP findings	Serial CTs with deep learning quantification
Lee et al., 2023 (26)	Asian health screening, age > 50	80% with ILA had radiologic progression	Median follow-up of 8 years	HR of 10.3 for progression in those with initially fibrotic ILA
Choe et al., 2025 (27)	Korean health screening, mean age 49 years	83.3% with ILA had radiologic progression	Median follow-up of 7 years	Progression defined by visual assessment
Chae et al., 2022 (82)	45 patients with surgical lung biopsy who also had incidental ILA	69% of subpleural fibrotic ILA found to have consistent or probable UIP histopathology	Median of 54 months for ILA progression	HR of 2.42 for progression in those with initially fibrotic ILA, median survival of 123 months
Salisbury et al., 2024 (58)	Unaffected family members of those with familial pulmonary fibrosis, mean age 53.2 years	65% progressed in those with initially mild ILA, 77% progressed in those with moderate ILA	Mean follow-up of 6.2 years	Progression defined radiologically for ILAs or transition to familial ILD
Salisbury et al., 2020 (34)	Unaffected first-degree relatives of those with familial interstitial pneumonia, mean age 53.1 years	63.3% with early/mild ILA progressed to ILD	5-year follow-up	Progression defined radiologically for ILAs or transition to familial ILD
Patel et al., 2023 (8)	Lung cancer screening cohort over 2 years, mean age 62.6 years	24.4% with ILA progression to ILD, 50% with any radiologic progression	Mean of 4.5 yrs. to ILD diagnosis	Presence of ILAs also associated with increased all-cause mortality
Jin et al., 2013 (19)	CT lung cancer screening population, mean age 61.5 years	Fibrotic ILA progressed in 37%, non-fibrotic ILA progressed in 11% and improved in 50%	2-year follow-up	ILAs categorized as subclinical and fibrotic vs. non-fibrotic
Araki et al., 2016 (83)	Framingham Heart Study, mean age of those with ILA progression 65 years	6% of ILA with progression	Serial CT scans completed on average 6 years apart, median follow-up of 4 years	ILA progression associated with greater FVC decline and increased mortality
Oh et al., 2024 (29)	Korean health screening cohort using automated quantification, mean age 49 years	23.5% ILA progression	Median follow-up of 6.5 years	Use of automated fibrosis score
Kawano-Dourado et al., 2020 (84)	RA cohort, mean age 65.6 in those with ILA/ILD	29% of ILA progressed	Mean follow-up of 4.4 years	Subpleural distribution and higher baseline extent associated with progression

### 3.2 Are there presenting clinical or radiologic findings that increase the risk of ILA progression to ILD?

Several factors have been linked to the progression of ILA to ILD including older age, smoking history, and extent and type of initial radiologic abnormalities (Table 3). Older age has been independently associated with the development and progression of ILA (29). Female sex was associated with the development or progression of ILD in one unadjusted analysis (29), while male sex has been associated with greater ILA risk. Lower forced vital capacity (FVC) appears to be associated with ILA progression, though those meeting restrictive criteria (<80%) may already be considered ILD, as described in one recent study (67). Reticulation, subpleural distribution, and higher baseline involvement (>10%) have been identified as presenting radiologic risk factors for ILD development (15). Traction bronchiectasis and honeycombing have been associated with ILA progression in one study (68), with eventual probable and definite usual interstitial pneumonia patterns also associated with increased mortality (69). Traction bronchiectasis may be an early non-dependent radiologic abnormality associated with developing fibrosis (OR 3.1 [1.3–7.3]) (70, 71). ILA progression appears to increase as traction bronchiectasis increases (72), with traction bronchiectasis also being associated with poorer survival (71). Other clinical factors associated with ILA progression include higher BMI (36) and elevated inflammatory markers such as erythrocyte sedimentation rate (ESR) (29). In those with familial history of pulmonary fibrosis, identifiable genetic abnormalities have been associated with increased risk of ILD and overall worse outcomes (24, 60).

**Table 3:** Risk factors associated with ILA progression.

Risk factor	Description/Details	Selected references
Older age	Increasing age consistently associated with higher risk of ILA development and progression	(5, 27, 83)
Smoking history	Ever-smoking status increases risk of progression	(27, 34, 58)
Male sex	Male sex associated with higher prevalence and progression risk	(5, 30)
Lower FVC% predicted	Reduced lung function at baseline increases risk	(83)
Subpleural fibrotic ILA (radiologic subtype)	Subpleural fibrotic pattern, reticulation, traction bronchiectasis, and honeycombing	(15, 22, 82, 85)
Environmental or occupational exposures	Ambient air pollution, self-reported exposures (e.g., birds, mold, aluminum, and lead)	(18, 34)
Genetic factors	MUC5B polymorphisms increases risk	(30, 34, 56, 83)
Short telomere length	Associated with increased risk of progression	(34, 57)
Elevated inflammatory markers and specific cell counts	Higher baseline white blood cell counts, monocyte counts, ESR, and rheumatoid factor associated with progression	(27, 86)
Comorbidities	OSA, GERD, and diabetes mellitus (in females) associated with ILA development and progression	(30, 36)

### 3.3 What is the overall prognosis or survival in those with ILA?

Individuals with ILAs generally experience worse prognosis and higher mortality rates compared to those without ILA. A recent meta-analysis described higher pooled mortality risk in individuals with ILAs (OR of 3.56 [2.19–5.81]) (5), with one study reporting 92 deaths per 1,000 person-years for those with ILA versus 61 deaths per 1,000 person-years for those without (hazard ratio (HR) 1.47 [1.05–2.05]) (33). Five-year mortality rates have been reported as 3.4% in those with ILA compared to 1.5% in unaffected individuals, with a 10-year mortality rate of 5.4% (29). Individuals who exhibited ILA progression had significantly worse survival than those with no ILA or ILA without progression (29, 69). Both fibrotic (HR 6.7 [3.7–12.2]) and nonfibrotic ILA (HR 5.3 [2.1–13.4]) are associated with higher mortality rates over 12 years than those without ILA (26).

### 3.4 What are current recommendations for the follow-up and monitoring of ILAs?

While screening in high-risk populations such as smokers or those with family history of pulmonary fibrosis may have greater yield, a single screening evaluation may not be sufficient for confirming the presence of early ILD or excluding future progression. Questions regarding optimal follow-up and its frequency remain, with current recommendations based on observed progression rates and associated risk factors, i.e., risk stratification. For individuals with newly detected ILA, follow-up scans at 2 to 3-year intervals may be appropriate for longitudinal monitoring. This is based on work by Park and colleagues who described a median time to ILA progression of 3.2 years, with median time to ILD diagnosis of 11.8 years (64). Patients with increased risk of progression such as subpleural fibrosis or traction bronchiectasis, may benefit from shorter follow-up intervals, such as every 12 months (1). Non-fibrotic ILAs may be managed expectantly with longer intervals and re-evaluation based on developing symptoms or clinical findings (2). After identification of suspected ILA, a first follow-up may be at 12 months or later to confirm persistence, before transitioning to every 3 years for long-term monitoring (73). Multidisciplinary team discussions in a clinic setting have also been suggested for assessing the underreporting of initial ILAs on incidental scans, relevance of background risk factors, and potential benefits or harms from additional testing vs. monitoring (74).

### 3.5 Is early treatment with antifibrotics or immunosuppression recommended in ILAs?

In about a quarter to a third of patients with radiologic progression of ILAs, findings may represent early ILD and an opportunity to pursue intervention before advanced fibrosis develops. For non-fibrotic ILAs or those without a basal-peripheral predominance, risk factor modification (e.g., smoking cessation, minimizing environmental exposures) and monitoring may be appropriate (2). It is unknown if non-fibrotic ILAs benefit from empiric immunosuppressants such as glucocorticoids or antimetabolites (mycophenolate or azathioprine) for slowing or reversing radiologic findings. Functional decline may be potentially impacted by earlier treatment of fibrotic ILAs before later stages of fibrosis develop, as theorized by one active study (75), though no current data exists on the early or pre-emptive use of anti-inflammatory or stop immunosuppressant agents in fibrotic or non-fibrotic ILAs. In summary, given the typically slow and heterogeneous natural history of ILAs, the absence of randomized data, and the potential harms of long-term pharmacologic therapy, current management appropriately emphasizes surveillance and timely identification of transition to ILD rather than pre-emptive treatment.

## 4 Discussion

### 4.1 Overview of current diagnosis and management

Current understanding of ILAs is significantly shaped by the Fleischner Society, which issued a position statement to standardize definitions and diagnostic criteria in 2020 (2). A recent 2025 ATS statement additionally summarizes the current literature and provides guidance on work up and management (3). ILAs are currently defined as bilateral nondependent radiologic abnormalities (ground-glass opacities, reticular patterns, architectural distortion, traction bronchiectasis, honeycombing, and/or non-emphysematous cysts) affecting ≥5% of a lung zone by visual estimate (3). Findings are incidental and subclinical, with radiologists being advised to identify or document them considering the high rate of missing or incorrect reporting described in prior studies (76, 77). Radiologists should compare initial findings with prior imaging if available, classify them into fibrotic or non-fibrotic subcategories, and recommend initial follow-up. Clinical evaluation may be recommended in those with more extensive presentations (often involving >10% of the lung with peripheral and basilar distribution) potentially representing ILD (1, 2). Being incidental findings by nature, much of the knowledge regarding prevalence, associated clinical and demographic findings, and specific radiologic abnormalities, is derived from subgroups undergoing screening programs for LC, coronary disease, or abdominal scans for non-pulmonary indications. Independent risk factors for the development and progression of ILAs overlap and include older age, male sex, current or prior smoking history, history of COPD or LC, and family history of pulmonary fibrosis. Other reported predictors of progression include more extensive radiologic abnormalities or fibrosis, elevated inflammatory markers, and BMI. Based on observational studies, more than half of ILAs progress radiologically within 3–5 years, with only a quarter to a third of those with progression subsequently being diagnosed with ILD. Current guidance suggests initial follow-up CT at 1 year, then every 3 years afterwards in those with initially fibrotic ILAs. Treatment of progressive ILAs is not recommended or known to be of benefit at this time, with current focus being on follow-up and monitoring for transition to ILD.

### 4.2 Controversies, challenges, and future directions

ILAs present a significant clinical challenge in terms of predicting and diagnosing potential ILD and evaluating the benefits of earlier treatment or risk factor modification for subsequent ILD outcomes. It is important to note that definitions provided by the Fleischner Society were originally meant for radiologist use to standardize the reporting of incidentally detected interstitial changes seen on CT scans obtained for other purposes (e.g., lung cancer screening, coronary imaging, nodule follow-up), and not imply or propose potential clinical implications or context. ILAs are essentially radiologic descriptors first, and not clinical diagnoses or syndromes. Overzealous expansion of this radiologic framework to a clinical context may lead to unnecessary investigations, referrals, and treatments in individuals who may never develop ILD — thereby increasing healthcare costs and patient anxiety and distress. Historical definitions of ILAs center around their incidental nature and the absence of clinical findings, yet some ILAs persist and progress, requiring multiple imaging studies to initially confirm and then monitor over time. If a proportion of ILAs go on to clinically significant ILD, should those at risk of developing ILAs (those with familial history or smokers for example) be screened for them in the first place? Perhaps all ILAs should be considered early ILD to justify a “screening” approach, even if only a portion go on to clinical ILD. Unfortunately, current understanding of ILAs is based on historically incidental presentations that may be limited to the groups in which they were observed. These findings may or may not be generalizable to all settings or individuals, considering differences in follow-up strategies (short vs. long), environmental or occupational exposures, and undetected genetic factors. There are also challenges with image-based distinction or thresholds for the transition of ILAs to ILD, noting current 5 and 10% cut-offs may be arbitrary. The best threshold for meeting ILD diagnosis, even in the absence of symptoms or functional abnormalities, remains an important issue to be explored as most studies describing transition to ILD reflected on “expert” clinical judgment without defining which symptomatic or functional criteria contributed to ILD diagnosis. Radiologic progression to typical ILD patterns may also suggest ILD even in the absence of symptoms or functional abnormalities, further challenging standardization and cut-offs for transition. Tools or approaches developed to determine which ILAs have greater likelihood of progression to ILD over what timeline, i.e., risk stratification, are core to efficient monitoring and early treatment plans. These may include the use of machine learning or artificial intelligence (13, 52, 78), multidimensional prediction models including radiomics with biomarkers such as polygenic risk scores and proteomic profiles (61, 79, 80), and redefining quantitative or qualitative thresholds for progression or more clinically relevant ILD diagnosis.

Lastly, there is currently no evidence to support empiric or directed treatment of progressive ILAs in terms of benefit or impacting ILD outcomes. The particular challenge is balancing subclinical presentations and slow rates of progression with the use of prolonged treatment courses involving pharmacologic agents of unclear efficacy in early or subclinical disease, yet significant adverse effects. Where the identification of ILAs may be clinically helpful is before the initiation of chemotherapy, radiation, or immune checkpoint inhibitors in patients with LC, as these patients appear to be at increased risk of treatment-related pneumonitis or fibrosis. Earlier recognition in this setting may allow for closer monitoring and revising management strategies. Otherwise, the detection or management of other potentially predictive or modifiable risk factors for ILA progression, such as pursuing broad genetic screening, smoking cessation, minimizing environmental or occupational exposures, reducing background inflammation, or encouraging weight loss, have yet to be proven as beneficial.

## 5 Conclusion

ILAs represent a spectrum of incidental subclinical radiologic findings that may precede or portend the development ILD. Evolving definitions and diagnostic criteria—shaped by recent consensus statements from the Fleischner Society and ATS—emphasize the importance of a standardized approach to reporting and risk stratification. ILAs are increasingly detected with prevalences varying by study population, age, smoking status, comorbidities, and pattern of follow-up. While a significant proportion of ILAs progress radiologically, only a subset transition to ILD, particularly those with initial fibrotic patterns, smoking history, and family history of pulmonary fibrosis. Current management focuses on risk stratification, longitudinal monitoring, targeted evaluation, and timely referral for those with eventual ILD. The role of preemptive antifibrotic or immunosuppressive therapy remains uncertain. Continued efforts to refine diagnostic thresholds for progression to ILD are necessary for optimizing treatment and patient outcomes.
